# Usage of cloud storage facilities by medical students in a low-middle income country, Sri Lanka: a cross sectional study

**DOI:** 10.1186/s12911-020-1029-z

**Published:** 2020-01-28

**Authors:** Samankumara Hettige, Eshani Dasanayaka, Dileepa Senajith Ediriweera

**Affiliations:** Centre for Health Informatics, Biostatistics and Epidemiology, Faculty of Medicine, University of Kelaniya, Ragama, Sri Lanka

**Keywords:** Cloud storage, Medical students, Undergraduates, Google drive, Dropbox

## Abstract

**Background:**

Cloud storage facilities (CSF) has become popular among the internet users. There is limited data on CSF usage among university students in low middle-income countries including Sri Lanka. In this study we present the CSF usage among medical students at the Faculty of Medicine, University of Kelaniya.

**Methods:**

We undertook a cross sectional study at the Faculty of Medicine, University of Kelaniya, Sri Lanka. Stratified random sampling was used to recruit students representing all the batches. A self-administrated questionnaire was given.

**Results:**

Of 261 (90.9%) respondents, 181 (69.3%) were females. CSF awareness was 56.5% (95%CI: 50.3–62.6%) and CSF usage was 50.8% (95%CI: 44.4–57.2%). Awareness was higher in males (*P* = 0.003) and was low in senior students. Of CSF aware students, 85% knew about Google Drive and 70.6% used it. 73.6 and 42.1% knew about Dropbox and OneDrive. 50.0 and 22.0% used them respectively. There was no association between CSF awareness and pre-university entrance or undergraduate examination performance. Inadequate knowledge, time, accessibility, security and privacy concerns limited CSF usage. 69.8% indicated that they would like to undergo training on CSF as an effective tool for education.

**Conclusion:**

CSF awareness and usage among the students were 56.5 and 50.8%. Google drive is the most popular CSF. Lack of knowledge, accessibility, concerns on security and privacy limited CSF usage among students. Majority were interested to undergo training on CSF and undergraduate Information Communication Technology (ICT) curricula should introduce CSF as effective educational tools.

## Background

John McCarthy first introduced the idea of Cloud Computing (CC) in 1961 [1], the term “cloud computing” is defined by the National Institutes of Standards and Technology (NIST) [2] as a model for enabling convenient, on-demand network access to a shared pool of configurable resources.

CC come in three service models: Software as a Service (SaaS), Platform as a Service (PaaS) and Infrastructure as a Service (IaaS). The SaaS aims to provide end users an access to applications such as: Gmail, Facebook, blog, etc. The PaaS offers services for the customers who can develop, test and run applications through the Internet such as GAE, Amazon Web Services and Microsoft Azure. The IaaS provides the users with resources such as servers, storage and computation facilities [3].

Clouds promise these benefits have been the reason for increasing adoption of cloud computing in many business areas already and in the healthcare domain in recent years [4]. For instance, the combination of CC and traditional mobile computing has resulted in the emergence of Mobile Cloud Computing in business sector [5]. One of Cloud promises in health sector is the possibility of handling the huge medical databases in order to improve the patient care by timely prediction with a good accuracy [6, 7]. Many more CC applications and capabilities are prominent in the other fields as well.

In CC dominant atmosphere in the world today, Cloud storage facilities (CSF) has gained popular over traditional storage media due to following advantages; free of charge availability by many providers, file synchronization facilities, file sharing facilities and reliability of services without worrying on data loss [8–12]. The CSF provide additional benefits for students other than saving their digital materials [12–14]. For example, students can take digital notes online and access them anytime and anyplace in a convenient way. These notes can be easily shared among colleagues. These help to avoid physical constraints face by students in accessing and sharing study materials. The CSF also facilitates collaborative work among students and increase productivity in group work [14].

Both students and university teachers use CSF to store teaching leaning and research materials. A previous study showed a higher demand for CSF in German higher education sector and 34% of higher education sector used cloud computing [15]**.** This is mainly due to the ease of access through any internet-enabled device [16], facilitate collaborative work [14] and CSF serves as backups and recovery solutions in hardware failures [17]. However the users of these public CSF have raised concerns on privacy invasion risks and data security breaches [14, 18]. Previous study done in Germany reported that 85% of university students used at least one CSF and Dropbox was the most popular CSF among them. Nearly 80% of students used CSF to store project work and teaching materials and 55% for other personal data [15].

There is limited data on CSF usage among university students in low middle income countries including Sri Lanka. This study was done to assess the knowledge, practice and attitude towards CSF among medical students at the Faculty of Medicine, University of Kelaniya. The existing literature does not provide sufficient evidence on the usage of CSF by medical students in low middle income countries. Hence our finding will be a unique contribution to world literature. Further, the finding will be a useful for administrators, policymakers and teachers in many higher education institutions in medicine, especially in developing countries in adaptation of ICT education in medical curricula. In Sri Lankan context, this study contributes for medical administrators to identify the future doctors’ attitude on trending technology like cloud base services.

### Related works

This section describes the previous literature in recent years which is related to the use in CSF by university students.

Several recent studies related the use of CSF by university students have been carried out. One large scale online survey [15] conducted by Meske et al., targeted more than 3000 participants including students (72%) as well as employees (28%) at the University of Muenster in Germany. The analysis of survey results indicated a high demand for cloud service solution in German higher education sector where the most of the students (85%) used at least one cloud service (employees: 73%). Students mainly used cloud services for educational purpose (project work -(83%) and teaching material - (78%)). Employees main use was to save work-related materials (78%). The most important reason for rejecting cloud storage services was security concerns (students: 64%; employees: 62%). The primary aim of this paper was to describe and present the main results of a preliminary large-scale survey on cloud services at the University of Muenster with more than 3000 participants in order to identify how the cloud service should be designed to be attractive for the target audience.

In another research [19] by Ashtari & Eydgahi examined how the engineering students at Eastern Michigan University accept and use the cloud services long after its adoption in the education process. The researchers used the Technology Acceptance Model (TAM) and Determinants of Perceived Ease of Use model to determine the CC adaptation by students.

A 97.5% of participants indicated that they are utilizing the cloud-based university class management application that enabled direct student access to Google Drive and other Google cloud suite. The majority of the students (97.5%) were utilizing at least two forms of cloud technology and 87.5% were using three or more applications. The reasons given by students for using cloud applications were: accessibility, the ability to share data, the low cost and the ability to back up files. The most common concerns were data privacy, fear of losing data, and difficulty of use. The researchers suggest that a combined model drawing from more aspects of internet technology will be more useful in further examinations of cloud computing adaptation.

Stantchev et al. [20] used the Technology Acceptance Model (TAM) to investigate the motivations that lead higher education students to replace several Learning Management System (LMS) services with cloud file hosting services in the field of information sharing and collaboration. Research findings extended previous research that has investigated the use of Dropbox to cover certain weaknesses of LMS within the higher education setting. The results showed that Dropbox receives better valuation than LMS for the three considered constructs: attitude toward using, perceived ease of use and perceived usefulness.

Another study based on first year medical students can be seen in the work of Peacock & Grande [21]. The main objective of their work was to present the results of effectively using a free Google cloud suite including Google Drive to manage and teach a first-year pathology course at Mayo Medical School in USA. The results demonstrated that Google cloud suite allowed faculty to build an efficient and effective classroom teaching and management system. 87% of participants responded positively in favor of Google Drive as a storage location for course materials. Ibrahim Arpaci et al. [12] investigated the adoption of cloud computing to achieve knowledge management using TAM. Researchers examined the cloud services involvement in knowledge creation and discovery, storage, sharing, and application among the students and concluded that the integration of cloud computing services into the educational settings may promote students’ academic performance, effectiveness, and efficiency by facilitating knowledge management mainly due to the cloud services that enable for students to access and synchronize their digital reference materials any time, from anywhere, and using any device. The Table 1 compares the similarities and differences in the current study with the other works stated in the Related Works section.

**Table 1:** Comparison with previous works

Author	Similarities and differences compared to the current study
Meske et al. in 2014 [15]	An Online survey that included whole student population and employees at the University of Muenster in Germany compared to the current study that used printed questionnaire to collect data from a sample of medical students. Both surveys focused on the use of CSF.
Ashtari & Eydgahi in 2017 [19]	The study sample was selected by inviting to 40 engineering students in a specified study setup. The objective was to find the students’ use and acceptance of CC by using TAM. The current study applied the stratified random sampling method to select the study sample from a medical faculty and attempted to find specifically the use of CSF.
Stantchev et al. in 2014 [20]	TAM was used to report weaknesses of several services of LMS over Dropbox cloud hosting service. Sample size was 121 students in computer science in final year and master level. Students involvement in Dropbox use as a CSF was the similarity found the two studies.
Peacock & Grande in 2016 [21]	This study involved the first year medical students in order to examine the possibility of effectively using a free Google cloud suite, including Google Drive to manage and teach a first-year pathology course. Medical students involved in the both studies. The current study specifically examine the CSF usage in the education process by the whole medical students.
Ibrahim et al. in 2017 [12]	221 Students in Information Technology (IT) subject stream who followed a training course on knowledge management and CC were involved in the study. The adaptation of cloud services in knowledge management was examined using TAM. Both studies focused on cloud services but on different research aspects.

### Hypotheses in the study

This study is designed to assess the following the hypotheses.

- The majority of the medical students do not use the CSF or underutilized in a situation where IT infrastructures are provided them free of charge.
- Students with prior experience of IT are the leading users of CSF.
- Students who perform well in previous and current exams use the CSF.

## Methodology

**Fig. 1:**
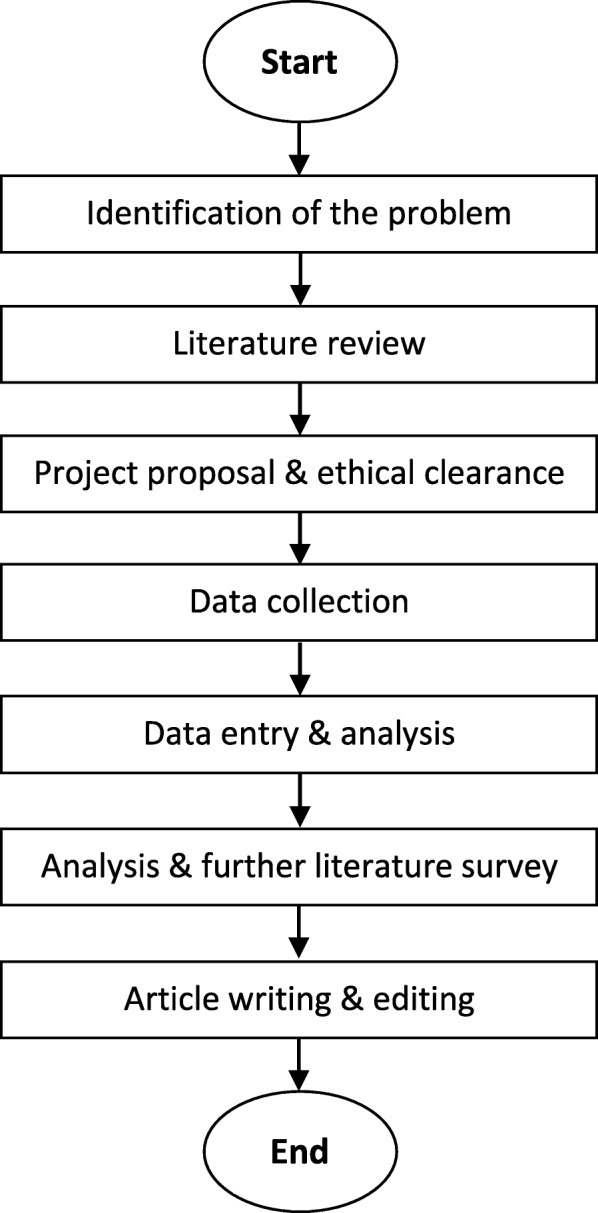
Flow chart of the research methodology

We undertook a cross sectional study at the Faculty of Medicine, University of Kelaniya, Sri Lanka. The research methodology depicts in the Fig. 1. The study was conducted from August 2016 to December 2016. A self-administrated questionnaire was used to collect data. There were five batches of medical students in the Faculty and stratified random sampling methods with proportional allocation was used to recruit students from each batch. Sample size was calculated to estimate the proportion of students who are aware of cloud storages using below formula and assumptions.

Sample size (SS) will be calculated using the following formula.

$$ \mathrm{Sample}\kern0.17em \mathrm{size}=\frac{{\mathrm{Z}}^2\ast \left(\mathrm{p}\right)\ast \left(1-\mathrm{p}\right)}{{\mathrm{c}}^2} $$

Where:

Z = Z value (i.e. Z = 1.96 for 95% confidence level).

p = percentage picking a choice, expressed as decimal (i.e. *p* = 0.5).

c = confidence interval, expressed as decimal (i.e. c = 0.05).

Hence, sample size was calculated as 384. Additional 10% was added as non-response bias (384 + 38 = 422). There were 903 total students in the Faculty and calculated sample size represented 47% of the population. Hence the sample size is more than 5% of the total population, we adopted finite population correction (FPC) to avoid over sampling.

Fine population correction (FPC):

$$ \mathrm{New}\;\mathrm{sample}\kern0.17em \mathrm{size}=\frac{{\mathrm{n}}_0}{\left(1+1/\mathrm{N}\left({\mathrm{n}}_0-1\right)\right)} $$

Where:

n_0_ = Initial sample size (i.e. 422).

N=Population size (i.e. 903).

Revised sample size was calculated as 287. Please refer the Additional file 1: Table S1 that provides the allocated number of students from each batch. Student’s name lists of each batch were obtained and the required number of students from each batch were selected using a random number generator.

Self-administered questionnaire was used to obtain data. The questionnaire had two parts. The first part of the questionnaire included student’s academic year, gender, results of the undergraduate medical examination including continuous assessments and Unit exams. Continuous assessments are formative examinations during the terms. Unit examinations are summative examinations and there are five Unit examinations with three subcomponents in Unit 3 (i.e. unit 3 a, b and c). Further, we obtained performance of General Certificate of Education Advanced Level (G.C.E. A/L) examination and grade 5 scholarship examination. G.C.E. A/L examination is the entrance examination to the State Universities of Sri Lanka and grade five scholarship is the selection examination for the prominent national schools in Sri Lanka. The second part of the questionnaire consisted of questions on CSF awareness and usage along with the frequency of usage. Of this part, one table was used to record the frequency of the CSF use.

Ethical approval for the study was obtained from the Faculty of Medicine, University of Kelaniya. All the medical students were over 18 years of age. At least one of authors participated in the data collection process. All the students were informed about the study in addition printed information, provided with the consent form to be completed by the participant. The informed written consent was obtained from those students who participated voluntarily in the study.

The following are the details explanation of the steps that show in the flowchart (Fig. 1).

- The computer labs of the faculty are used for practical classes in medicine and teaching ICT. The students are not allowed to use external devices in teaching lab due to security reasons. This makes an unpleasant situation for some students. Students can easily overcome this problem using the free cloud storage. The issue is whether the students are aware of cloud storage or not. No previous data was available in the faulty in this regard.
- Literature survey proved the lack of research in the use of CSF by medical students, specially from developing countries.
- The faculty encourages to have the ethical clearance for researches that involves students. Hence the project proposal was submitted to faculty ethical clearance committee to have the approval for the research.
- The questionnaires that was revised by the ethic committee were distributed among the selected students. A sample of 287 students were selected after applying the finite population correction (FPC) to recalculate the sample size that was selected with the stratified random sampling methods with proportional allocation.
- Data was entered and verified in the REDCap software system.
- R statistical package was used to perform descriptive analysis on data.
- Did the literature surveys to compare our findings with the world. Data analysis and literature surveys were repeated as required.
- Started the article writing in the following order: Methodology, Data analysis, Discussion, Conclusion, abstract and Introduction.

## Results

The statistical analysis was done using R version 3.5.3. The average CSF awareness and usage were calculated with confident intervals. Pearson’s Chi-square test statistics were checked for statistical differences between genders. Spearman’s rank correlation coefficient was used to determine the connection, if any between CSF awareness and exam performance. The trend in the awareness of CSF between academic years was checked using the Generalized linear model.

### Description of the study sample

We distributed 287 questionnaires and 261 (90.9%) students responded. There were 181 (69.3%) female students in the sample. Number of students who responded to the questionnaire from first year to final year as follows: 49 (18.8%), 55 (21.1%), 51 (19.5%), 59 (22.6%) and 47 (18.0%). 13 students did not respond to the question on awareness of CSF and we removed them from the analysis. These students belonged to all academic years and respective numbers were 2,3,2,3 and 3 from first year to final year. We present the results of 248 respondents in the subsequent analysis.

### Awareness of CSF

Of 248 students, 140 indicated that they were aware of CSF indicating 56.5% (95%CI: 50.3–62.6%) CSF awareness among medical students at the Faculty of Medicine, University of Kelaniya. CSF awareness was higher in males (71.1, 95%CI: 60.9–81.2%) compared to females (50.0, 95%CI: 42.5–57.5%) (*P* = 0.003). CSF awareness among the first year students (72.3, 95%CI: 60.0–85.1%) was higher than the final year students (40.9, 95%CI: 26.4–55.4%). Generalized linear model fitting showed a significant decrease in CSF awareness in the senior students (odds ratio = 0.74 (95%CI: 0.62–0.90) per each advancing batch).

Among 140 of students who were aware of CSF, highest awareness was observed for Google Drive (85.0%). Second and third highest awareness were for Dropbox (73.6%) and OneDrive (42.1%). 63 (45.0%) students were already aware of CSF before entering to the University (Table 2).

**Table 2:** Demographic characteristics of the participants

Characteristics	Aware of cloud storage (*N* = 140) Number (56.5%)	Not aware of cloud storage (*N* = 108) Number (43.5%)	*P* value
Gender			
Males	54 (71.1%)	22 (28.9%)	0.003*
Females	86 (50.0%)	86 (50.0%)	
Academic year			
First year	34 (72.3%)	13 (27.7%)	0.003^†^
Second year	32 (61.5%)	20 (38.5%)	
Third year	26 (53.1%)	23 (46.9%)	
Fourth year	30 (53.6%)	26 (46.4%)	
Final year (fifth year)	18 (40.9%)	26 (59.1%)	
When did the students get to know			
Before entering to the Faculty	63 (45.0%)		
After entering to the faculty and learnt during the IT practical sessions	25 (17.6%)		
After entering to the faculty but not learn from the IT practical sessions	47 (33.6%)		
Not-answered	5 (3.6%)		

*Pearson’s Chi squared test; ^†^Linear logistic/Generalized linear model

### Usage of CSF

Of 140 who were aware of CSF, 126 (90.0%) actually used these facilities. Hence, the CSF usage among all the students at the Faculty was 50.8% (95%CI: 44.4–57.2%). Male students showed a relatively higher CSF usage (62.5, 95%CI: 51.9–73.1%) compared to female students (47.0, 95% CI: 39.7–54.2%), although the difference was not significant.

From the students who were aware of Google Drive (85.0%), 12.1% had accessed it daily and 20.0% had accessed it more than once a week. Second and third highest awareness were for Dropbox (73.6%) and OneDrive (42.1%). From them more than 15% had never accessed them. Although students were aware of iCloud and Amazon, majority had never accessed them (Table 3).

**Table 3:** Awareness and usage patterns of the participants for the top five cloud storages

Name	Awareness Number (%)	Daily	More than once a week	More than once a month	Less than once a month	Never	Not answered
Google Drive	119 (85.0%)	17 (12.1%)	28 (20.0%)	31 (22.1%)	23 (16.4%)	13 (9.3%)	28 (20.0%)
Dropbox	103 (73.6%)	8 (5.7%)	13 (9.3%)	21 (15.0%)	28 (20.0%)	25 (17.9%)	45 (32.1%)
OneDrive	59 (42.1%)	5 (3.6%)	9 (6.4%)	10 (7.1%)	11 (7.8%)	26 (18.6%)	79 (56.4%)
iCloud	53 (37.9%)	7 (5.0%)	7 (5.0%)	6 (4.2%)	11 (7.8%)	30 (21.4%)	79 (56.4%)
Amazon	33 (23.6%)	1 (0.7%)	2 (1.4%)	2 (1.4%)	3 (2.1%)	32 (23.6%)	100 (71.4%)

Among the students who were aware of CSF, 79 (56.4%) had used cloud facility to transfer electronic files and 64 (45.7%) students used cloud storage to save educational materials. 36 (25.7%) had synchronized their files with CSF. Further, 109 (77.9%) students mentioned that cloud storage is useful for educational purposes and 19 (13.5%) students in view that CSF is little or no use for educational purpose.

### Awareness of CSF and exam performance

There was no difference in the Z score of G.C.E. A/L between students who were aware and not aware of CSF (median score: 1.96 (IQR: 1.92–2.00) versus 1.98 (IQR: 1.92–2.00) respectively, *P* = 0.251). Similarly, there was no difference in grade five scholarship results between students who were aware and not aware of cloud storages (median score: 151 (IQR: 140.0–162.0) versus 151.0 (IQR: 143.0–163.0) respectively, *P* = 0.434).

We could not elicit a correlation between CSF awareness and the medical exam performances of students. Spearman’s rank correlation coefficient for continuous assessments as follows; continuous assessment 1 = − 0.107 (*P* = 0.107); continuous assessment 2 = − 0.028 (*P* = 0.701). For unit examinations it was; unit 1 = 0.032 (*P* = 0.668); unit 2 = 0.129 (*P* = 0.090); unit 3a = − 0.001 (*P* = 0.997); unit 3b = 0.084 (*P* = 0.431); unit 3c = 0.144 (*P* = 0.179); unit 4 = 0.146 (*P* = 0.375); unit 5 = − 0.188 (*P* = 0.257).

### Limiting factors of using CSF

Of 140 CSF aware students, 55 (39.3%) and 28 (20.0%) mentioned that they do not have adequate time and knowledge to use CSF respectively. Limiting factors for using CS included lack of accessibility 45(32.1%), concerns on security 37 (26.4%) and privacy 32 (22.9%).

### Interest to learn more about CSF

Of total students, 35 (14.1%) student did not want to use CSF and 173 (69.8%) indicated that they would like to learn CSF as an effective tool for education. This included 72 (76.6%) of students who were not previously aware of CSF and 101 (84.9%) students who were already aware of CSF. 40 (16.1%) students did not wish to have a training on CSF and this included 22 (23.4%) students who were not aware of CSF and 18 (15.1%) who were aware of CSF.

## Discussion

Among the students at the Faculty of Medicine, University of Kelaniya, 56.5% (95%CI: 50.3–62.6%) were aware of CSF and 50.8% (95%CI: 44.4–57.2%) used CSF. Our results showed a lower CSF usage among local medical students compare to western countries [15, 19]**.** It is important to note that Faculty of Medicine has provided unlimited Wi-Fi internet access to the students. All the students had opportunity to learn ICT during the first year of the degree program and among the CSF aware students only 17.6% had learnt CSF during the ICT lessons. These ICT lessons were not compulsory for students and low students’ attendance were observed for ICT lessons. Male students showed a higher awareness compared to females and newer students showed higher awareness compared to older students reflecting higher technology penetration among males and newer students. However, there was no CSF usage difference between males and female students.

Of those who were aware of CSF, nearly 50% of students used CSF to store educational materials and 25% had used synchronize facility to create backups [15, 19]. Further 50% students had used CSF to transfer electronic files [19]. Google Drive was the most popular CSF among the students, 12% accessed it daily and 20% used it more than once a week [21, 22]. Dropbox and OneDrive were the second and third popular CSF among students. However, more than 17% of them had never accessed them. Although students were aware of iCloud and Amazon, the majority had never accessed them. This pattern was different to a previous study where Dropbox was the most popular CSF among students [10, 15, 20].

We could not elicit a difference in Z score at G.C.E. Advance Level results nor grade five scholarship results among students who were aware and not aware of CSF. G.C.E. Advance Level is the entrance examination to state universities and grade five-scholarship examination is the selection examination for the prominent national schools in the country. This shows that CSF awareness does not depend on the nature of the school which students attended, whether those schools were equipped with ICT facilities or not, nor educational performance before entering to the University. We could not elicit a correlation between CSF usage and examination performance in the University that shows students who were familiar with ICT tools were not in advantage over others.

Main limiting factors for using CSF were lack of accessibility and concerns on security and privacy [23, 24]. Further students mentioned lack of time and knowledge hindered CSF usage [19]. The majority of those who were aware of CSF in view that these facilities can be used for educational purposes and 13.6% expressed that CSF is no or little use for academic activities. Out of all who participated, 82% were in favor of having a training on CSF and this was 76.6% among who did not know CSF. This reflects the requirement of ICT education throughout the medical curriculum rather than limiting it to the first year. Students often neglect ICT lessons during the first year due to workload of other subjects and do not understand the importance of ICT in continuous professional development.

### Limitations of the study

Following limitations are in this study; higher percentage of female students were recruited to the study as we adopted stratified random sampling. This is due to the fact that there were higher percentage of females in among the students. Unit examination results were available only for the senor students as junior students were still had not reached to the Unit examination level. This study included students from only one state medical faculty of the country.

## Conclusion

Our survey results showed that CSF awareness and usage among the students were 56.5 and 50.8%. Google drive is the most popular CSF followed by Dropbox and OneDrive. Lack of knowledge, accessibility, concerns on security and privacy limited CSF usage among students. Majority were interested to undergo training on CSF and undergraduate ICT curricula should introduce CSF as effective educational tools. We emphasize the requirement of the ICT exposure in medical education to overcome the technological challenges face by future doctors. Future research, not limited one institution is encouraged to have more validated results but it is challenging to convince the importance of this kind of research in every medical institution.

## Supplementary information

**Additional file 1: Table S1.** Number of students allocated to each batch in the study sample.

## Data Availability

Data are available on request to the authors.

## Abbreviations

CC: Cloud computing
CSF: Cloud storage facilities
ICT: Information communication technology
LMS: Learning management system
TAM: Technology acceptance model
