# Supplementary material for: Usage of cloud storage facilities by medical students in a low-middle income country, Sri Lanka: a cross sectional study
Source: BMC Med Inform Decis Mak. 2020 Jan 28;20:10. doi: 10.1186/s12911-020-1029-z (PMC6986067; doi:10.1186/s12911-020-1029-z)
Supplement: Supplementary file 1 — Additional file 1: Table S1. Number of students allocated to each batch in the study sample. [file 12911_2020_1029_MOESM1_ESM.docx]

**Supplementary table 1**

| Academic year | Total number of students | Selected number of students |
| --- | --- | --- |
| First year | 163 | 52 |
| Second year | 174 | 55 |
| Third year | 168 | 54 |
| Fourth year | 199 | 63 |
| Final year | 199 | 63 |
| Total | 903 | 287 |
